# Comment on Fernandez Martin et al. Analgesia for Upper Abdominal Surgery, a Scoping Review of the Current Fascial Plane Block Techniques. *J. Clin. Med.* 2025, *14*, 8632

**DOI:** 10.3390/jcm15083151

**Published:** 2026-04-21

**Authors:** Reginald Edward

**Affiliations:** Department of Anaesthesia, Austin Health, Melbourne, VIC 3844, Australia; reginald.edward@austin.org.au

Thank you for the opportunity to respond. I welcome the discussion on the evolving role of regional analgesia in upper abdominal surgery [1]. However, I believe the framing that epidural analgesia has been “superseded” warrants clarification.

My position is not that epidural analgesia persists due to lack of awareness of alternatives, but rather that it has been prematurely de-prioritised without adequate exploration of modernised delivery strategies. Much of the contemporary critique conflates the limitations of traditional epidural practice with intrinsic limitations of neuraxial analgesia itself.

Several commonly cited disadvantages of thoracic epidural analgesia, including hypotension, urinary retention, delayed mobilisation, and increased ward workload, are largely attributable to dose and delivery choices, not to neuraxial analgesia per se [2,3,4]. Analgesic-dose regimens using low-concentration local anaesthetic, particularly when combined with contemporary delivery modes such as programmed intermittent bolus, reduce sympathetic blockade, improve longitudinal spread, and lower total local anaesthetic exposure [5,6,7]. These approaches are physiologically sound and well supported in neuraxial literature, yet remain under-represented in perioperative pathways.

In contrast, while ultrasound-guided fascial plane blocks represent an important advance, the current evidence base supports their role primarily as adjuncts rather than substitutes. Their effects are predominantly somatic, often short-lived, and inconsistently associated with improvements in patient-centred recovery outcomes when compared with optimised systemic multimodal analgesia [8,9,10]. To date, no high-quality evidence demonstrates equivalence to a well-functioning thoracic epidural for major open upper abdominal surgery [11,12].

I am concerned that institutional preference has increasingly been shaped by operational convenience and risk aversion, rather than comparative effectiveness. This shift has occurred alongside declining investment in neuraxial expertise, ward-level support, and protocol refinement. In many settings, abandonment has preceded optimisation.

My intent is therefore not to advocate epidurals as a default, but to argue for intellectual honesty in acknowledging trade-offs. Where epidural analgesia is contraindicated or impractical, alternative strategies should be used transparently, recognising that they may offer inferior analgesic coverage for certain surgical pain trajectories. Reframing this compromise as replacement risks obscuring clinically meaningful differences.

I suggest that a more constructive approach is to re-evaluate epidural analgesia within a modern ERAS framework, focusing on refined dosing, delivery, and governance, while positioning fascial plane blocks appropriately as complementary tools rather than equivalents. This distinction is essential if analgesic strategy is to remain aligned with physiology rather than fashion.

I appreciate the editor’s engagement with this debate and hope this response contributes to a more nuanced and evidence-aligned discussion.
